# Oral neglect as a marker of broader neglect: a cross-sectional investigation of orodental consultation letter of leukemic admitted patients in Iran

**DOI:** 10.1186/s12903-021-01775-x

**Published:** 2021-08-21

**Authors:** Fatemeh Owlia, Amin Ansarinia, Hassanali Vahedian Ardakani

**Affiliations:** 1 Department of Oral and Maxillofacial Medicine, School of Dentistry, Shahid Sadoughi University of Medical Sciences, Yazd, Iran; 2 Dental Students’ Research Committee, Department of Orthodontics, School of Dentistry, Isfahan University of Medical Sciences, Isfahan, Iran; 3 Department of Oncology, Shahid Sadoughi University of Medical Sciences, Yazd, Iran

**Keywords:** Leukemia, Referral, Consultation, Oral manifestations

## Abstract

**Background:**

Leukemia is the main malignant hematologic disease of children with different oral manifestations and clinical features. Attention to the oral manifestations is essential for better management. This study assessed the oral and dental consultations conducted in the admitted leukemic patients of an Iranian teaching hospital.

**Methods:**

In this descriptive cross-sectional study, medical records of patients admitted in Yazd Shahid Sadoughi Hospital were evaluated. Records of 300 patients with leukemia were randomly selected. Data including demographic information on age, sex, type of oral problems, prescribed instructions and leukemia type were extracted from archived records and registered on the checklist. Finally, Data were analyzed by SPSS17 using Chi-square test.

**Results:**

The results showed the average age ± SD of patients were 24.36 ± 23.91 with a range of 4 days to 86 years. Among 300 files, 167 belonged to males (55.7%) and 133 pertained to females (44.3%). The most prevalent type of underlying disease was ALL (Acute lymphocytic leukemia) with the frequency of 180 persons (60%). Only 12 (4%) of patients were referred to a specialist due to oral problems. Of all cases of consultation requests, 75% received consultation because of dental problems and 25% for mucosal problems. There was a statistically significant difference between age and consultation request (P = 0.002).

**Conclusions:**

According to the results of this study, orodental consultation request rate for admitted leukemic patients was low. Pediatric hematologist and oncologists to some extent had more interest to consult due to oral and dental problems rather than others.

**Supplementary Information:**

The online version contains supplementary material available at 10.1186/s12903-021-01775-x.

## Background

Oral health, as a mirror of body health, could be influenced by different problems and impacts the quality of life. Leukemia is the most common type of cancer among children in the Middle East [1] and the sixth common type of cancer in Iran [2]. It happens when there is a deficiency in proliferation or evolution of white blood cells (WBCs) [1]. Accumulation of these cancerous cells outside the bone marrow, leads to the formation of masses either in oral mucosa or vital body organs [3, 4]. Acute leukemia is the most common type of malignancy among children. Leukemia has varying manifestations in different organs and oral cavity. These oral problems are observed in the majority of patients and may be the first sign that could lead to diagnosis [5, 6].

Oral involvement in acute leukemia is reported in 91% of patients and 85% of patients who are on chemotherapy and in 100% of cases who are treated by radiotherapy [7–10]. Gingival hyperplasia, mucosal wounds and bleeding [5–7], and lymphadenopathy are some signs of orofacial problems [7].

The aim of multidisciplinary consultation is to have two physicians with different specialties to cooperate in order to achieve the best solution to the problem and better management of patients in appropriate time. The request for medical consultation reflects the diagnosis and communication skills and also professionalism of a physician [11]. Good communication is necessary to a high-quality consultation process. A large number of health staff have not passed any courses on consultation; besides, most of them faced with a situation of consultation request [12]. Failure in the consultation may lead to late diagnosis, inappropriate prescription and unnecessary polypharmacy, unnecessary laboratory tests, and medical expenses [13].

Alibhai et al. [14] concluded that among cancer patients who referred to specialized centers, the rate of referral was higher significantly in those under 19 years; also, there was a clear negative correlation between age of the patients and medical consultation requests. In other words, the rate of medical consultation was higher in children.

Moreover, a study by Villa et al. [15] in America demonstrated that leukemic patients affected by oral problems were visited by different physicians before referring to an oral medicine specialist. This is due to the low level of information about this specialty among physicians and ordinary people.

Considering the review of studies, it seems that a little amount of information is available about medical consultation in patients with hematologic cancer due to oral complications [16, 17]; timely diagnosis of oral complications caused by leukemia has a vital role in prognosis and patients’ quality of life. This study evaluated the prevalence of oral and dental consultations conducted in the admitted leukemic patients in the Oncology Department of Yazd Shahid Sadoughi Hospital during February and March, 2017.

## Methods

This descriptive cross-sectional study was carried out on 300 leukemic patients’ archived files with a history of admission in Yazd Shahid Sadoughi Hospital during February and March, 2017, were randomly selected according to the random number table. This study was approved by the Committee of Ethics in Human Research at Yazd Shahid Sadoughi University of Medical Sciences (ID#: IR.SSU.REC.1396.191). Simple census sampling method was used to select the subjects. Considering P = %10 obtained in the pilot study and a confidence level of 95% (α = 0.05) and accuracy of 4% (d = 0.04), the sample size was determined as 217. According to statisticians’ opinion and approval of local ethical committee, to obtain more reliable and stronger statistical results, the sample size was increased to 300. With all legal dimensions, as a routine treatment program of the academic hospital, an informed consent consisting the permission of using medical records and oral examination separately was obtained from admitted adult patients or their parents and/or legal guardian of the patients younger than 18 years old, at the time of admission.

Data composed of demographic information on age, sex, type of oral problems, prescribed instructions and leukemia type, and were extracted from archived records and registered on the checklist.

Data were analyzed by SPSS17 using Chi-square test (P < 0.05). All methods were conducted in accordance with the ethical standards of the declaration of Helsinki.

## Results

Among 300 files, there were 167 males (55.7%) and 133 females (44.3%). The average age ± SD of patients was 24.36 ± 23.91 ranging between 4 days and 86 years (Table 1).

**Table 1:** Relative frequency distribution of study patients according to demographic variables

Variable	N	%
*Age*		
≤ 9	126	42
10–29	72	24
≥ 30	102	34
*Sex*		
Male	167	55.7
Female	133	44.3

The average age ± SD of 16 medical specialists who were consulted was 47.91 ± 3.88 with a range of 34 to 65 years. Among them, there were 14 males and 2 females. The profile of doctors who prescribed dental consultations is displayed in Table 2. Among 16 medical specialists, only 3 medical specialists (2 pediatric hematologist and oncologists and 1 adult hematologist and Oncologist) had consulted with dentists (Table 2).

**Table 2:** Profile and number of consultation request of doctors participating in the present study

Specialty	Number of doctors	Number of consultation request
Pediatric hematology and oncology	2	2
Adult hematology and oncology	2	1
Others	12	0

As it is shown in Table 3, ALL (Acute lymphocytic leukemia) was the most common type of leukemia with the frequency of 60% of patients.

**Table 3:** Frequency distribution of consultation request rate according to underlying disease type and age of patients

Variable	Consoling request rate	Frequency	p^a^
*Underlying disease type*			
ALL	10 (83%)	180 (60%)	
AML	1 (8.3%)	70 (23.3%)	0.065
CLL	0 (0%)	26 (8.7%)	
CML	0 (0%)	20 (6.7%)	
Others	1 (8.3%)	4 (1.3%)	
Total	12 (100%)	300 (100%)	
*Patients, age*			
≤ 9	11 (91.6%)	126 (42%)	0.002*
10–29	0 (0%)	72 (24%)	
≥ 30	1 (8.4%)	102 (34%)	
Total	12 (100%)	300 (100%)	

*ALL* acute lymphocytic leukemia, *AML* acute myeloid leukemia, *CLL* chronic lymphocytic leukemia, *CML* chronic myeloid leukemia

^a^Differences between patients with and without consultation request according to type of underlying disease and patients, age were evaluated using the χ^2^ test. The level of statistical significance was set at P < 0.05

The result of this analysis showed that only 12 (4%) patients were referred to a specialist due to oral problems. Of all cases of consultation requests, 75% consulted because of dental problems and 25% for mucosal problems.

The frequency distribution of consultation requests according to the patient's sex demonstrated that among 12 patients who requested for consultation, 5 patients (41.6%) were male and 7 patients (58.4%) were female. Chi-Square test indicated that there was not any statistically significant difference based on sex.

Based on type of hematologic cancer, the highest number of consultation requests were related to ALL with 83.3%. Furthermore, there was not any consultation request in patients affected by CLL (Chronic Lymphocytic Leukemia) and CML (Chronic Myeloid Leukemia) (Table 3).

There is no statically significant difference according to type of hematologic cancer (P value = 0.065).

The distribution of consultation request as a function of the patient's age are listed in Table 3.

As it is shown in Table 3, there was a significant statistical difference between age and consultation request (P = 0.002) and the highest number was requested in children up to 9.

Oral problems were evaluated in 6 groups, 108 patients (36%) had at least one oral problem, while in 64% of cases there were no data related to oral problems, unfortunately (Table 4).

**Table 4:** Relative frequency of oral signs and symptoms and prescription for oral problems based on patients’ files

Variable	N	%
*Oral signs and symptoms*		
Oral ulcerations	47	15.7
Mucosal bleeding	34	11.3
Swelling	31	10.3
Toothache	11	3.7
Burning sensation	13	4.3
Others	10	3.3
*Descriptive instruction*		
Anti-microbial	48	16
Anti-inflammatory	65	21.7
Combination drug	73	24.3
Others	28	9.3

The prescribed instructions were classified in 4 groups and no medicine was prescribed for 176 of cases (58.6%); besides, for 124 of cases (41.4%), at least one category of medicine in the list was prescribed (Table 4).

## Discussion

The average age ± SD of patients was 24.36 ± 23.91 with a range 4 days to 86 years. Among 300 patients, there were 167 males and 133 females. Also, among 16 medical specialists, there were 14 males and 2 females. The most prevalent type of underlying disease was ALL.

Oral complications related to the chemotherapy were seen in more than 75% of cases [18]. On the other hand, in many cases, oral complications in early stages leads to the diagnosis of leukemia [5, 6]; thus, dentists have a vital role in 33% of AML diagnosis [19].

In this study, the frequency of oral complications of studied cases was only 36% which is similar to the frequency of the study carried out by Nakhostin et al. [17] (39.7%); however, in clinical studies, the frequency of oral complications was determined higher than this study [5, 6, 20]. Nakhostin et al. [17] believed that the reason of low prevalence of oral complications in their study was the issue of not registering them in patients’ files. Our results are consistent with the study above. In the present study, the patients' hospital files were evaluated for oral complications. They were documented by medical specialists or medical interns, not oral medicine specialists. The differences between this study and previous ones could be attributed to ignoring some oral manifestations by medical physicians. Higher percent of prescribed instructions (41.4%) compared to registered oral complications (36.6%) confirms this. It is suggested that the data related to orodental complications should be recorded in hospitals by oral medicine specialists to omit data collection bias.

In the present study, at least one drug was prescribed to overcome oral complications in 41.4% of patients. Besides, 83.3% of patients who were referred to a dentist received prescription; nonetheless, in the study by Navarro et al. [21], only 14.8% of patients referred to an oral medicine specialist received prescription. This demonstrates that in this investigation physicians prefer to treat oral and dental complications without consultation with a dentist; by the way, in this study, unlike other studies which recommend single drug regimen for mucosal problems [19, 22, 23], the most common treatment was the combined mouthwash (a mixture of Diphene hydramine, Nystatin, Aluminum-Mg syrup and Normal Saline). It should be mentioned that the composition of this mouthwash was different from other mouthwashes [7, 24]. These differences in the treatment plan of oral complications in cancer patients are consistent with the results of a study by Glenny et al. [25]. According to their study, despite the existence of specific guidelines in this field, clinicians of 6 medical centers in the United Kingdom manage oral complications in different ways. These differences may be due to patient cooperation or physicians’ preference, following oral routine care, staff awareness, and time limitations. However, in that study, the most common prescription used for oral problems was multi drug therapy.

The most frequent oral complication caused by leukemia in this study was, to some extent, the same as another Iranian study by Ghasemzadeh et al. [26]. In their study, the most common oral complication was bleeding, but in this study, bleeding ranked second after mucosal wounds. Also, the least frequent problem in both studies was dental problems.

In the present study, frequency of oral complications and referral of patients were 36% and 4%, respectively. Dela Cruz et al. [27] reported that 78% of primary care clinicians were inclined to refer their patients to a dentist if they faced symptoms of primary caries or high risk patient. Nevertheless, in this study, medical specialists’ performance was assessed. The oral consultation requests were 11%. The big difference between two studies indicates that Iranian medical specialists are unaware of the importance of consultation with oral medicine specialists.

Another reason pertained was the lack of residents of dentistry or oral medicine specialists in hospitals for consultation while Lockhart et al. [13] mentioned the presence of 4 full time dentists as one of important factors in referral to dentists. On the other hand, according to studies by Hashemipour et al. [28] and Tomlinson et al. [29], the long distance between dental centers and the studied hospitals is another reason for the lack of consultation request for oral problems.

According to Dela Cruz et al. [27], most pediatric primary care providers had some difficulties in referring their patients to dentists. They believed increasing access to dental services is necessary to reduce the problem, and suggested some solutions such as increasing the number of dental graduates and recruiting more dentists in the public care system.

Indeed, in the study by Collard et al. [30], various health care professionals were unable to provide dental services; in the study by Lewis et al. [31], 46% of pediatricians took part in oral health-related activities and less than 25% of pediatricians had received oral health education during university course or continuing education.

It is better to consult with an oral medicine specialist for all of admitted patients who are at risk of any oral complications [16, 27]. Some reasons for not doing dental consultation promptly, could be time limitations, lack of proper knowledge of the appropriate time for dental intervention and lack of resources [25]. It is noteworthy that all the consultation requests were carried out by hematologist and oncologists; of course, 91.6% were undertaken by pediatric hematologist and oncologists. This finding confirmed the results of a study by Glenny et al. [25]. They mentioned a big difference in the provision of dental services to children and adults with cancer.

Consultation request due to oral problems has been ignored in other fields of medicine throughout the world. This may be the result of lack of awareness and attitude towards oral medicine among medical specialists. The level of awareness of medical specialists and other healthcare staff toward the referral and consultation due to oral problems with dentists was low in previous studies [15, 28, 32–37]. Meanwhile, medical and dental students demonstrated a positive attitude towards awareness of the collaboration between medical and dental practice in Hong Kong [38]. The necessity of education in this field is unanimous. However, some studies have shown that the level of knowledge of medical specialist about different fields of dentistry had no effect on the amount of consultation and referrals to them [27].

In the present study, only 25% of dental consultations were related to mucosal problems and the remaining 75% were associated with dental problems, while in the study by Lockhart et al. [13], nearly 72% of referrals to dentists were due to mucosal problems and 28% for dental problems. These significant differences between two studies may be attributed to low knowledge of medical physicians toward oral medicine or interest in managing oral problems by themselves. Unfortunately, researches have shown that in other studies, less attention has been paid to the health of the oral mucosa and only dental problems such as caries or dental malformations were considered a good reason for dental consultation. Nevertheless, neglecting the oral health and oral complications in leukemic patients could be life-threatening [16, 27, 31, 39].

In the present study, the rate of consultation requests varied significantly depending on age of patients. Indeed, 91.6% of referrals pertained to children up to 9 years old. It may be due to the greater number of children affected by acute leukemia which causes more oral manifestations. Higher mitotic rate and thinner mucosal epithelium in children, make them vulnerable to mucosal defects and oral problems. On the other hand, children's frequent restlessness and inability to eat, make physicians look for solutions to the problem [40]. Alibhai et al. [14] showed a significant negative correlation between patient's age and referral request rate in patients affected by AML. In this study, similar to the study by Dela Cruz et al. [27], other patient characteristics did not show any clear relationship with referral rate.

To overcome the problems and obstacles of consultation with dentists, the presence of a number of oral medicine residents in hospitals, a proper educational curriculum, spreading information to increase the knowledge of patients and healthcare staff are recommended.

Clinicians should bear in mind that the role of oral health in the quality of life of cancer patients is vital. It would be interesting to open a line of research on this topic in future.

To our knowledge, this is the first report showing ignoring consultation request due to the existing oral and dental problems of admitted leukemic patients in Iran. It would be the basis of need assessment in health promotion planning and reducing morbidity and mortality of cancer.

## Limitations

One of the limitations of this study was sampling from only a single medical center. Of course, considering this was the only teaching hospital for leukemic patients’ admission in Yazd, this limitation could be ignored. Another limitation of the study was incomplete patients' files. To overcome this problem, all incomplete files would be randomly replaced with other files.

The novelty of this study includes evaluating the performance of different medical specialists in dental consultation at the teaching hospital in Yazd, center of Iran. Unfortunately, this subject has been currently ignored in developing countries.

## Conclusion

Our findings showed that orodental consultation request rate for admitted leukemic patients was low; pediatric hematologist and oncologists to some extent had more interest to consult due to oral and dental problems rather than others. Regarding the high prevalence of oral problems in leukemic patients, timely consultation with oral medicine specialists could play a vital role in quality of life and survival of involved patients.

## Supplementary Information

**Additional file 1**. Raw data.

## Data Availability

All data generated or analyzed during this study are included in this published article (and its raw analysis: Additional file 1).

## Abbreviations

ALL: Acute lymphocytic leukemia
AML: Acute myeloid leukemia
CLL: Chronic lymphocytic leukemia
CML: Chronic myeloid leukemia
