# Supplementary material for: Oral neglect as a marker of broader neglect: a cross-sectional investigation of orodental consultation letter of leukemic admitted patients in Iran
Source: BMC Oral Health. 2021 Aug 21;21:413. doi: 10.1186/s12903-021-01775-x (PMC8380362; doi:10.1186/s12903-021-01775-x)
Supplement: Supplementary file 1 — Additional file 1. Raw data. [file 12903_2021_1775_MOESM1_ESM.doc]

**Oral neglect as a marker of broader neglect: a cross-sectional investigation of orodental**  **consultation letter of leukemic admitted patients in Iran**

Authors: Fatemeh Owlia ^1,2^, Amin Ansarinia ^*3^, Hassanali Vahedian Ardakani ^4^

1. Associate professor, Department of Oral and Maxillofacial Medicine, School of Dentistry, Shahid Sadoughi University of Medical Sciences, Yazd, Iran.
2. Social Determinants of Oral Health Research Center, Shahid Sadoughi University of Medical Sciences, Yazd, Iran.
3. Postgraduate student, orthodontics department, school of dentistry, Isfahan university of medical sciences, Isfahan, Iran
4. Associate Professor, Department of Oncology, Shahid Sadoughi University of Medical Sciences, Yazd, Iran

*Corresponding author: Dr Amin Ansarinia, Postgraduate student, orthodontics department, school of dentistry, Isfahan university of medical sciences, Isfahan, Iran. Email: ansar.ssu@gmail.com Tel: +989135233006 – 035 32336353

|  | number | agePatient | gender |  | a  g  e  O  n  c  o  l  o  .  .  . | univOncolo... | bimeh |
| --- | --- | --- | --- | --- | --- | --- | --- |
| 1 | 1 | 37.00 |  | 1 | 63 | 4 | 1 |
| 2 | 2 | 12.00 |  | 1 | 44 | 1 | 4 |
| 3 | 3 | 0.03 |  | 1 | 44 | 1 | 4 |
| 4 | 4 | 0.30 |  | 1 | 44 | 1 | 4 |
| 5 | 5 | 3.00 |  | 1 | 44 | 1 | 2 |
| 6 | 6 | 2.00 |  | 1 | 44 | 1 | 2 |
| 7 | 7 | 4.00 |  | 1 | 44 | 1 | 2 |
| 8 | 8 | 4.00 |  | 2 | 44 | 1 | 1 |
| 9 | 9 | 4.00 |  | 2 | 44 | 1 | 1 |
| 10 | 10 | 15.00 |  | 2 | 44 | 1 | 1 |
| 11 | 11 | 5.00 |  | 1 | 44 | 1 | 1 |
| 12 | 12 | 12.00 |  | 1 | 44 | 1 | 1 |
| 13 | 13 | 15.00 |  | 2 | 44 | 1 | 1 |
| 14 | 14 | 5.00 |  | 1 | 44 | 1 | 1 |
| 15 | 15 | 5.00 |  | 2 | 44 | 1 | 1 |
| 16 | 16 | 12.00 |  | 1 | 44 | 1 | 1 |
| 17 | 17 | 11.00 |  | 1 | 44 | 1 | 1 |
| 18 | 18 | 11.00 |  | 2 | 44 | 1 | 1 |
| 19 | 19 | 6.00 |  | 2 | 44 | 1 | 1 |
| 20 | 20 | 2.00 |  | 1 | 44 | 1 | 1 |
| 21 | 21 | 3.00 |  | 1 | 44 | 1 | 1 |
| 22 | 22 | 4.00 |  | 2 | 44 | 1 | 1 |
| 23 | 23 | 11.00 |  | 2 | 44 | 1 | 1 |
| 24 | 24 | 6.00 |  | 1 | 44 | 1 | 1 |
| 25 | 25 | 4.00 |  | 1 | 44 | 1 | 1 |
| 26 | 26 | 14.00 |  | 2 | 44 | 1 | 1 |
| 27 | 27 | 3.00 |  | 1 | 44 | 1 | 1 |
| 28 | 28 | 4.00 |  | 1 | 44 | 1 | 1 |
| 29 | 29 | 11.00 |  | 2 | 44 | 1 | 1 |
| 30 | 30 | 2.00 |  | 1 | 44 | 1 | 1 |
| 31 | 31 | 11.00 |  | 2 | 44 | 1 | 1 |
| 32 | 32 | 5.00 |  | 1 | 44 | 1 | 1 |
| 33 | 33 | 13.00 |  | 2 | 44 | 1 | 1 |
| 34 | 34 | 2.00 |  | 1 | 44 | 1 | 1 |
| 35 | 35 | 3.00 |  | 1 | 44 | 1 | 1 |

|  | illness |  | DarkhastM... | elateErja | z  a  k  h  m |  | khoonrizi | tavarom |
| --- | --- | --- | --- | --- | --- | --- | --- | --- |
| 1 |  | 2 | 2 | . |  | 1 | 1 | 2 |
| 2 |  | 1 | 2 | . |  | 2 | 2 | 2 |
| 3 |  | 2 | 2 | . |  | 1 | 2 | 2 |
| 4 |  | 2 | 2 | . |  | 1 | 2 | 2 |
| 5 |  | 1 | 2 | . |  | 2 | 2 | 2 |
| 6 |  | 1 | 2 | . |  | 2 | 2 | 2 |
| 7 |  | 1 | 2 | . |  | 2 | 2 | 2 |
| 8 |  | 1 | 2 | . |  | 2 | 2 | 1 |
| 9 |  | 1 | 2 | . |  | 2 | 2 | 2 |
| 10 |  | 1 | 2 | . |  | 2 | 2 | 2 |
| 11 |  | 1 | 2 | . |  | 2 | 2 | 2 |
| 12 |  | 1 | 2 | . |  | 2 | 2 | 2 |
| 13 |  | 1 | 2 | . |  | 2 | 2 | 2 |
| 14 |  | 1 | 2 | . |  | 2 | 2 | 2 |
| 15 |  | 1 | 2 | . |  | 2 | 2 | 2 |
| 16 |  | 1 | 2 | . |  | 2 | 2 | 2 |
| 17 |  | 1 | 2 | . |  | 2 | 2 | 1 |
| 18 |  | 1 | 2 | . |  | 2 | 2 | 2 |
| 19 |  | 1 | 2 | . |  | 2 | 2 | 2 |
| 20 |  | 1 | 2 | . |  | 2 | 2 | 2 |
| 21 |  | 1 | 2 | . |  | 2 | 2 | 2 |
| 22 |  | 1 | 2 | . |  | 2 | 1 | 2 |
| 23 |  | 1 | 2 | . |  | 2 | 2 | 2 |
| 24 |  | 1 | 1 | 2 |  | 2 | 2 | 2 |
| 25 |  | 1 | 1 | 2 |  | 2 | 1 | 2 |
| 26 |  | 1 | 2 | . |  | 1 | 2 | 2 |
| 27 |  | 1 | 1 | 2 |  | 1 | 1 | 1 |
| 28 |  | 1 | 2 | . |  | 2 | 2 | 2 |
| 29 |  | 1 | 2 | . |  | 2 | 2 | 2 |
| 30 |  | 1 | 2 | . |  | 2 | 1 | 2 |
| 31 |  | 1 | 2 | . |  | 1 | 2 | 2 |
| 32 |  | 1 | 2 | . |  | 2 | 2 | 2 |
| 33 |  | 1 | 2 | . |  | 2 | 2 | 2 |
| 34 |  | 1 | 2 | . |  | 2 | 1 | 2 |
| 35 |  | 1 | 2 | . |  | 2 | 2 | 2 |

|  | dandani | symptom | TazahorSayer | a  n  t  i  m  i  c  r  o  b  i  a  l | zedeEltehab | asareTarkibi |
| --- | --- | --- | --- | --- | --- | --- |
| 1 | 1 | 2 | 2 | 1 | 2 | 2 |
| 2 | 2 | 2 | 2 | 2 | 1 | 2 |
| 3 | 2 | 2 | 2 | 2 | 2 | 1 |
| 4 | 2 | 2 | 2 | 1 | 1 | 2 |
| 5 | 2 | 2 | 2 | 1 | 1 | 2 |
| 6 | 2 | 2 | 2 | 1 | 2 | 2 |
| 7 | 2 | 2 | 2 | 2 | 1 | 2 |
| 8 | 2 | 2 | 2 | 2 | 1 | 2 |
| 9 | 2 | 2 | 2 | 2 | 1 | 2 |
| 10 | 2 | 2 | 2 | 2 | 1 | 2 |
| 11 | 2 | 2 | 2 | 2 | 1 | 2 |
| 12 | 2 | 2 | 2 | 2 | 1 | 2 |
| 13 | 2 | 2 | 2 | 2 | 1 | 2 |
| 14 | 2 | 2 | 2 | 2 | 1 | 2 |
| 15 | 2 | 2 | 2 | 2 | 1 | 2 |
| 16 | 2 | 2 | 2 | 2 | 1 | 2 |
| 17 | 2 | 2 | 2 | 2 | 1 | 2 |
| 18 | 2 | 2 | 2 | 2 | 1 | 2 |
| 19 | 2 | 2 | 2 | 2 | 2 | 1 |
| 20 | 2 | 2 | 2 | 2 | 2 | 2 |
| 21 | 2 | 2 | 2 | 2 | 1 | 1 |
| 22 | 2 | 2 | 2 | 2 | 1 | 2 |
| 23 | 2 | 2 | 2 | 2 | 1 | 2 |
| 24 | 2 | 2 | 2 | 2 | 2 | 2 |
| 25 | 2 | 2 | 1 | 2 | 2 | 1 |
| 26 | 2 | 2 | 2 | 1 | 2 | 1 |
| 27 | 2 | 1 | 2 | 2 | 2 | 1 |
| 28 | 2 | 2 | 2 | 2 | 2 | 2 |
| 29 | 2 | 2 | 2 | 2 | 2 | 2 |
| 30 | 2 | 2 | 2 | 2 | 2 | 2 |
| 31 | 2 | 2 | 2 | 2 | 1 | 1 |
| 32 | 2 | 2 | 2 | 2 | 1 | 2 |
| 33 | 2 | 2 | 2 | 2 | 2 | 2 |
| 34 | 2 | 2 | 2 | 2 | 2 | 2 |
| 35 | 2 | 2 | 2 | 2 | 2 | 2 |

| Dastoorola... | |
| --- | --- |
| 1 | 2 |
| 2 | 2 |
| 3 | 2 |
| 4 | 1 |
| 5 | 2 |
| 6 | 2 |
| 7 | 2 |
| 8 | 2 |
| 9 | 2 |
| 10 | 2 |
| 11 | 2 |
| 12 | 2 |
| 13 | 2 |
| 14 | 2 |
| 15 | 2 |
| 16 | 2 |
| 17 | 2 |
| 18 | 2 |
| 19 | 2 |
| 20 | 2 |
| 21 | 2 |
| 22 | 2 |
| 23 | 2 |
| 24 | 2 |
| 25 | 2 |
| 26 | 2 |
| 27 | 2 |
| 28 | 2 |
| 29 | 2 |
| 30 | 2 |
| 31 | 2 |
| 32 | 2 |
| 33 | 2 |
| 34 | 2 |
| 35 | 2 |

|  | number | agePatient | gender |  | a  g  e  O  n  c  o  l  o  .  .  . | univOncolo... | bimeh |
| --- | --- | --- | --- | --- | --- | --- | --- |
| 36 | 36 | 3.00 |  | 1 | 44 | 1 | 1 |
| 37 | 37 | 1.00 |  | 1 | 44 | 1 | 1 |
| 38 | 38 | 5.00 |  | 1 | 44 | 1 | 1 |
| 39 | 39 | 1.00 |  | 1 | 44 | 1 | 1 |
| 40 | 40 | 1.00 |  | 2 | 44 | 1 | 1 |
| 41 | 41 | 0.75 |  | 1 | 44 | 1 | 1 |
| 42 | 42 | 8.00 |  | 2 | 44 | 1 | 1 |
| 43 | 43 | 8.00 |  | 2 | 44 | 1 | 1 |
| 44 | 44 | 1.00 |  | 2 | 44 | 1 | 1 |
| 45 | 45 | 11.00 |  | 2 | 44 | 1 | 1 |
| 46 | 46 | 2.00 |  | 2 | 44 | 1 | 1 |
| 47 | 47 | 5.00 |  | 1 | 44 | 1 | 1 |
| 48 | 48 | 8.00 |  | 2 | 44 | 1 | 7 |
| 49 | 49 | 9.00 |  | 2 | 44 | 1 | 7 |
| 50 | 50 | 8.00 |  | 2 | 44 | 1 | 3 |
| 51 | 51 | 1.00 |  | 2 | 44 | 1 | 3 |
| 52 | 52 | 2.00 |  | 1 | 44 | 1 | 3 |
| 53 | 53 | 2.00 |  | 1 | 44 | 1 | 3 |
| 54 | 54 | 4.00 |  | 2 | 44 | 1 | 3 |
| 55 | 55 | 7.00 |  | 2 | 44 | 1 | 3 |
| 56 | 556 | 11.00 |  | 1 | 44 | 1 | 3 |
| 57 | 557 | 3.00 |  | 1 | 44 | 1 | 3 |
| 58 | 58 | 3.00 |  | 1 | 44 | 1 | 3 |
| 59 | 59 | 13.00 |  | 2 | 44 | 1 | 3 |
| 60 | 60 | 10.00 |  | 1 | 44 | 1 | 5 |
| 61 | 61 | 9.00 |  | 1 | 44 | 1 | 5 |
| 62 | 62 | 2.00 |  | 1 | 44 | 1 | 7 |
| 63 | 63 | 10.00 |  | 2 | 41 | 2 | 3 |
| 64 | 64 | 56.00 |  | 1 | 53 | 4 | 1 |
| 65 | 65 | 74.00 |  | 2 | 53 | 4 | 1 |
| 66 | 66 | 74.00 |  | 2 | 53 | 4 | 1 |
| 67 | 67 | 17.00 |  | 2 | 50 | 1 | 2 |
| 68 | 68 | 43.00 |  | 2 | 50 | 1 | 2 |
| 69 | 69 | 59.00 |  | 1 | 50 | 1 | 1 |
| 70 | 70 | 15.00 |  | 2 | 50 | 1 | 1 |

|  | illness |  | DarkhastM... | elateErja | z  a  k  h  m |  | khoonrizi | tavarom |
| --- | --- | --- | --- | --- | --- | --- | --- | --- |
| 36 |  | 1 | 2 | . |  | 2 | 2 | 1 |
| 37 |  | 1 | 2 | . |  | 2 | 2 | 2 |
| 38 |  | 1 | 2 | . |  | 2 | 2 | 2 |
| 39 |  | 2 | 2 | . |  | 2 | 2 | 2 |
| 40 |  | 2 | 2 | . |  | 2 | 2 | 2 |
| 41 |  | 2 | 2 | . |  | 2 | 2 | 2 |
| 42 |  | 2 | 2 | . |  | 2 | 2 | 2 |
| 43 |  | 2 | 2 | . |  | 1 | 2 | 2 |
| 44 |  | 5 | 2 | . |  | 2 | 2 | 1 |
| 45 |  | 5 | 2 | . |  | 1 | 2 | 1 |
| 46 |  | 4 | 2 | . |  | 2 | 2 | 2 |
| 47 |  | 5 | 1 | 1 |  | 1 | 1 | 2 |
| 48 |  | 1 | 2 | . |  | 2 | 2 | 2 |
| 49 |  | 1 | 1 | 1 |  | 2 | 2 | 1 |
| 50 |  | 1 | 2 | . |  | 2 | 2 | 2 |
| 51 |  | 1 | 2 | . |  | 2 | 2 | 1 |
| 52 |  | 1 | 2 | . |  | 2 | 2 | 2 |
| 53 |  | 1 | 2 | . |  | 2 | 1 | 2 |
| 54 |  | 1 | 2 | . |  | 2 | 2 | 2 |
| 55 |  | 1 | 2 | . |  | 2 | 2 | 2 |
| 56 |  | 1 | 2 | . |  | 2 | 2 | 1 |
| 57 |  | 1 | 2 | . |  | 2 | 2 | 1 |
| 58 |  | 1 | 2 | . |  | 2 | 2 | 2 |
| 59 |  | 2 | 2 | . |  | 2 | 2 | 1 |
| 60 |  | 1 | 2 | . |  | 2 | 2 | 2 |
| 61 |  | 1 | 2 | . |  | 2 | 2 | 2 |
| 62 |  | 1 | 2 | . |  | 2 | 2 | 2 |
| 63 |  | 1 | 2 | . |  | 2 | 2 | 2 |
| 64 |  | 1 | 2 | . |  | 1 | 1 | 2 |
| 65 |  | 3 | 2 | . |  | 2 | 2 | 2 |
| 66 |  | 3 | 2 | . |  | 2 | 2 | 2 |
| 67 |  | 1 | 2 | . |  | 2 | 1 | 2 |
| 68 |  | 2 | 2 | . |  | 2 | 2 | 2 |
| 69 |  | 1 | 2 | . |  | 2 | 2 | 2 |
| 70 |  | 1 | 2 | . |  | 2 | 2 | 2 |

|  | dandani | symptom | TazahorSayer | a  n  t  i  m  i  c  r  o  b  i  a  l | zedeEltehab | asareTarkibi |
| --- | --- | --- | --- | --- | --- | --- |
| 36 | 2 | 2 | 1 | 1 | 1 | 1 |
| 37 | 2 | 2 | 2 | 2 | 2 | 2 |
| 38 | 2 | 2 | 2 | 2 | 2 | 2 |
| 39 | 2 | 2 | 2 | 1 | 1 | 1 |
| 40 | 2 | 2 | 2 | 1 | 1 | 1 |
| 41 | 2 | 2 | 2 | 2 | 1 | 2 |
| 42 | 2 | 2 | 2 | 2 | 1 | 1 |
| 43 | 2 | 2 | 2 | 2 | 1 | 1 |
| 44 | 2 | 2 | 2 | 2 | 1 | 1 |
| 45 | 2 | 2 | 2 | 2 | 1 | 1 |
| 46 | 2 | 2 | 2 | 2 | 1 | 2 |
| 47 | 2 | 2 | 2 | 2 | 2 | 2 |
| 48 | 2 | 2 | 2 | 2 | 1 | 2 |
| 49 | 1 | 2 | 2 | 2 | 1 | 1 |
| 50 | 2 | 2 | 2 | 2 | 2 | 2 |
| 51 | 2 | 2 | 2 | 2 | 1 | 1 |
| 52 | 2 | 2 | 2 | 2 | 2 | 2 |
| 53 | 2 | 2 | 2 | 1 | 1 | 2 |
| 54 | 2 | 2 | 2 | 2 | 2 | 2 |
| 55 | 2 | 2 | 2 | 2 | 2 | 2 |
| 56 | 2 | 2 | 2 | 2 | 2 | 2 |
| 57 | 2 | 2 | 2 | 2 | 1 | 1 |
| 58 | 2 | 2 | 2 | 2 | 1 | 2 |
| 59 | 2 | 2 | 2 | 1 | 2 | 2 |
| 60 | 2 | 2 | 2 | 2 | 1 | 2 |
| 61 | 2 | 2 | 2 | 2 | 2 | 2 |
| 62 | 2 | 2 | 2 | 2 | 2 | 2 |
| 63 | 2 | 2 | 2 | 2 | 2 | 2 |
| 64 | 2 | 2 | 2 | 2 | 2 | 2 |
| 65 | 2 | 2 | 2 | 2 | 2 | 2 |
| 66 | 2 | 2 | 2 | 2 | 2 | 2 |
| 67 | 2 | 2 | 2 | 2 | 2 | 1 |
| 68 | 2 | 2 | 2 | 2 | 2 | 2 |
| 69 | 2 | 2 | 2 | 1 | 2 | 2 |
| 70 | 2 | 2 | 2 | 2 | 2 | 2 |

| Dastoorola... | |
| --- | --- |
| 36 | 2 |
| 37 | 2 |
| 38 | 2 |
| 39 | 2 |
| 40 | 2 |
| 41 | 2 |
| 42 | 2 |
| 43 | 2 |
| 44 | 2 |
| 45 | 1 |
| 46 | 2 |
| 47 | 2 |
| 48 | 2 |
| 49 | 1 |
| 50 | 2 |
| 51 | 2 |
| 52 | 2 |
| 53 | 2 |
| 54 | 2 |
| 55 | 2 |
| 56 | 2 |
| 57 | 2 |
| 58 | 2 |
| 59 | 2 |
| 60 | 2 |
| 61 | 2 |
| 62 | 2 |
| 63 | 2 |
| 64 | 2 |
| 65 | 2 |
| 66 | 2 |
| 67 | 1 |
| 68 | 2 |
| 69 | 2 |
| 70 | 2 |

|  | number | agePatient | gender |  | a  g  e  O  n  c  o  l  o  .  .  . | univOncolo... | bimeh |
| --- | --- | --- | --- | --- | --- | --- | --- |
| 71 | 71 | 15.00 |  | 2 | 50 | 1 | 1 |
| 72 | 72 | 29.00 |  | 1 | 50 | 1 | 1 |
| 73 | 73 | 34.00 |  | 1 | 50 | 1 | 1 |
| 74 | 74 | 35.00 |  | 1 | 50 | 1 | 1 |
| 75 | 75 | 68.00 |  | 1 | 50 | 1 | 1 |
| 76 | 76 | 20.00 |  | 2 | 50 | 1 | 1 |
| 77 | 77 | 25.00 |  | 2 | 50 | 1 | 1 |
| 78 | 78 | 62.00 |  | 1 | 50 | 1 | 1 |
| 79 | 79 | 26.00 |  | 2 | 50 | 1 | 1 |
| 80 | 80 | 30.00 |  | 1 | 50 | 1 | 1 |
| 81 | 81 | 51.00 |  | 1 | 50 | 1 | 1 |
| 82 | 82 | 20.00 |  | 2 | 50 | 1 | 1 |
| 83 | 83 | 32.00 |  | 2 | 50 | 1 | 1 |
| 84 | 84 | 43.00 |  | 2 | 50 | 1 | 1 |
| 85 | 85 | 50.00 |  | 2 | 50 | 1 | 1 |
| 86 | 86 | 77.00 |  | 1 | 50 | 1 | 1 |
| 87 | 87 | 62.00 |  | 2 | 50 | 1 | 1 |
| 88 | 88 | 79.00 |  | 1 | 50 | 1 | 1 |
| 89 | 89 | 79.00 |  | 1 | 50 | 1 | 1 |
| 90 | 90 | 59.00 |  | 2 | 50 | 1 | 1 |
| 91 | 91 | 51.00 |  | 1 | 50 | 1 | 1 |
| 92 | 92 | 51.00 |  | 1 | 50 | 1 | 1 |
| 93 | 93 | 51.00 |  | 1 | 50 | 1 | 1 |
| 94 | 94 | 33.00 |  | 1 | 50 | 1 | 1 |
| 95 | 95 | 35.00 |  | 1 | 50 | 1 | 1 |
| 96 | 96 | 35.00 |  | 1 | 50 | 1 | 1 |
| 97 | 97 | 35.00 |  | 1 | 50 | 1 | 3 |
| 98 | 98 | 39.00 |  | 2 | 50 | 1 | 3 |
| 99 | 99 | 20.00 |  | 1 | 50 | 1 | 3 |
| 100 | 100 | 22.00 |  | 1 | 50 | 1 | 3 |
| 101 | 101 | 50.00 |  | 1 | 50 | 1 | 3 |
| 102 | 102 | 50.00 |  | 1 | 50 | 1 | 3 |
| 103 | 103 | 38.00 |  | 1 | 50 | 1 | 6 |
| 104 | 104 | 39.00 |  | 1 | 50 | 1 | 6 |
| 105 | 105 | 26.00 |  | 2 | 50 | 1 | 6 |

|  | illness |  | DarkhastM... | elateErja | z  a  k  h  m |  | khoonrizi | tavarom |
| --- | --- | --- | --- | --- | --- | --- | --- | --- |
| 71 |  | 1 | 2 | . |  | 1 | 2 | 2 |
| 72 |  | 1 | 2 | . |  | 2 | 1 | 2 |
| 73 |  | 2 | 2 | . |  | 1 | 2 | 2 |
| 74 |  | 2 | 2 | . |  | 1 | 2 | 2 |
| 75 |  | 2 | 2 | . |  | 1 | 1 | 2 |
| 76 |  | 2 | 2 | . |  | 1 | 2 | 2 |
| 77 |  | 2 | 2 | . |  | 2 | 2 | 2 |
| 78 |  | 2 | 2 | . |  | 2 | 2 | 2 |
| 79 |  | 2 | 2 | . |  | 1 | 2 | 2 |
| 80 |  | 2 | 2 | . |  | 2 | 2 | 2 |
| 81 |  | 2 | 2 | . |  | 2 | 2 | 1 |
| 82 |  | 2 | 2 | . |  | 2 | 2 | 2 |
| 83 |  | 2 | 2 | . |  | 2 | 2 | 2 |
| 84 |  | 2 | 2 | . |  | 2 | 2 | 2 |
| 85 |  | 2 | 2 | . |  | 2 | 1 | 2 |
| 86 |  | 2 | 2 | . |  | 2 | 2 | 2 |
| 87 |  | 3 | 2 | . |  | 2 | 2 | 2 |
| 88 |  | 3 | 2 | . |  | 2 | 1 | 2 |
| 89 |  | 3 | 2 | . |  | 2 | 2 | 2 |
| 90 |  | 3 | 2 | . |  | 2 | 2 | 2 |
| 91 |  | 4 | 2 | . |  | 2 | 2 | 2 |
| 92 |  | 4 | 2 | . |  | 2 | 1 | 2 |
| 93 |  | 4 | 2 | . |  | 2 | 1 | 2 |
| 94 |  | 4 | 2 | . |  | 2 | 2 | 2 |
| 95 |  | 3 | 2 | . |  | 2 | 2 | 2 |
| 96 |  | 3 | 2 | . |  | 2 | 2 | 2 |
| 97 |  | 1 | 2 | . |  | 1 | 2 | 2 |
| 98 |  | 1 | 2 | . |  | 2 | 2 | 2 |
| 99 |  | 2 | 2 | . |  | 2 | 2 | 2 |
| 100 |  | 2 | 2 | . |  | 1 | 2 | 2 |
| 101 |  | 2 | 2 | . |  | 2 | 1 | 2 |
| 102 |  | 2 | 2 | . |  | 2 | 2 | 2 |
| 103 |  | 1 | 2 | . |  | 1 | 2 | 1 |
| 104 |  | 1 | 2 | . |  | 1 | 2 | 2 |
| 105 |  | 2 | 2 | . |  | 1 | 2 | 2 |

|  | dandani | symptom | TazahorSayer | a  n  t  i  m  i  c  r  o  b  i  a  l | zedeEltehab | asareTarkibi |
| --- | --- | --- | --- | --- | --- | --- |
| 71 | 2 | 1 | 2 | 2 | 2 | 1 |
| 72 | 2 | 2 | 2 | 2 | 2 | 2 |
| 73 | 2 | 2 | 2 | 2 | 2 | 1 |
| 74 | 1 | 2 | 2 | 2 | 2 | 2 |
| 75 | 2 | 2 | 2 | 2 | 2 | 2 |
| 76 | 2 | 2 | 1 | 1 | 2 | 2 |
| 77 | 2 | 2 | 2 | 2 | 2 | 2 |
| 78 | 2 | 2 | 2 | 2 | 2 | 2 |
| 79 | 2 | 2 | 2 | 1 | 2 | 2 |
| 80 | 2 | 2 | 2 | 1 | 2 | 2 |
| 81 | 2 | 2 | 2 | 2 | 2 | 2 |
| 82 | 2 | 2 | 2 | 2 | 1 | 1 |
| 83 | 2 | 2 | 2 | 2 | 2 | 2 |
| 84 | 2 | 2 | 2 | 2 | 2 | 2 |
| 85 | 2 | 2 | 2 | 2 | 2 | 2 |
| 86 | 2 | 2 | 2 | 2 | 2 | 2 |
| 87 | 2 | 2 | 2 | 2 | 2 | 2 |
| 88 | 2 | 1 | 2 | 2 | 2 | 2 |
| 89 | 2 | 2 | 2 | 2 | 2 | 2 |
| 90 | 2 | 2 | 2 | 2 | 2 | 1 |
| 91 | 2 | 2 | 2 | 2 | 2 | 2 |
| 92 | 2 | 2 | 2 | 2 | 2 | 2 |
| 93 | 2 | 2 | 1 | 2 | 2 | 2 |
| 94 | 2 | 2 | 2 | 2 | 2 | 2 |
| 95 | 1 | 2 | 2 | 2 | 2 | 2 |
| 96 | 2 | 2 | 2 | 2 | 2 | 2 |
| 97 | 2 | 2 | 2 | 2 | 2 | 1 |
| 98 | 2 | 2 | 2 | 1 | 2 | 1 |
| 99 | 2 | 2 | 2 | 1 | 2 | 1 |
| 100 | 2 | 2 | 2 | 2 | 2 | 2 |
| 101 | 2 | 2 | 2 | 1 | 2 | 2 |
| 102 | 2 | 2 | 1 | 2 | 2 | 2 |
| 103 | 2 | 1 | 2 | 1 | 2 | 1 |
| 104 | 2 | 2 | 2 | 2 | 2 | 2 |
| 105 | 2 | 2 | 2 | 2 | 2 | 1 |

|  | Dastoorola... |
| --- | --- |
| 71 | 2 |
| 72 | 2 |
| 73 | 2 |
| 74 | 2 |
| 75 | 2 |
| 76 | 2 |
| 77 | 2 |
| 78 | 2 |
| 79 | 2 |
| 80 | 1 |
| 81 | 2 |
| 82 | 1 |
| 83 | 2 |
| 84 | 2 |
| 85 | 2 |
| 86 | 2 |
| 87 | 2 |
| 88 | 2 |
| 89 | 2 |
| 90 | 2 |
| 91 | 2 |
| 92 | 2 |
| 93 | 2 |
| 94 | 2 |
| 95 | 2 |
| 96 | 2 |
| 97 | 2 |
| 98 | 2 |
| 99 | 2 |
| 100 | 2 |
| 101 | 2 |
| 102 | 2 |
| 103 | 2 |
| 104 | 2 |
| 105 | 2 |

|  | number | agePatient | gender |  | a  g  e  O  n  c  o  l  o  .  .  . | univOncolo... | bimeh |
| --- | --- | --- | --- | --- | --- | --- | --- |
| 106 | 106 | 33.00 |  | 2 | 50 | 1 | 6 |
| 107 | 7 | 65.00 |  | 2 | 50 | 1 | 7 |
| 108 | 8 | 21.00 |  | 2 | 50 | 1 | 5 |
| 109 | 9 | 67.00 |  | 1 | 50 | 1 | 5 |
| 110 | 10 | 23.00 |  | 2 | 50 | 1 | 5 |
| 111 | 11 | 59.00 |  | 2 | 50 | 1 | 5 |
| 112 | 12 | 69.00 |  | 1 | 50 | 1 | 5 |
| 113 | 13 | 53.00 |  | 2 | 50 | 1 | 7 |
| 114 | 14 | 1.00 |  | 2 | 42 | 4 | 3 |
| 115 | 15 | 22.00 |  | 2 | 37 | 2 | 1 |
| 116 | 16 | 79.00 |  | 2 | 37 | 2 | 1 |
| 117 | 17 | 60.00 |  | 1 | 65 | 4 | 5 |
| 118 | 18 | 86.00 |  | 2 | 50 | 2 | 1 |
| 119 | 19 | 29.00 |  | 1 | 45 | 2 | 1 |
| 120 | 20 | 78.00 |  | 2 | 45 | 2 | 1 |
| 121 | 21 | 36.00 |  | 1 | 45 | 2 | 7 |
| 122 | 22 | 64.00 |  | 1 | 65 | 4 | 1 |
| 123 | 23 | 40.00 |  | 1 | 65 | 4 | 7 |
| 124 | 24 | 13.00 |  | 2 | 53 | 4 | 1 |
| 125 | 25 | 51.00 |  | 2 | 34 | 4 | 1 |
| 126 | 26 | 51.00 |  | 2 | 34 | 4 | 1 |
| 127 | 24 | 26.00 |  | 2 | 45 | 1 | 4 |
| 128 | 28 | 30.00 |  | 1 | 45 | 1 | 2 |
| 129 | 29 | 72.00 |  | 2 | 45 | 1 | 2 |
| 130 | 30 | 63.00 |  | 1 | 45 | 1 | 1 |
| 131 | 31 | 42.00 |  | 1 | 45 | 1 | 1 |
| 132 | 32 | 60.00 |  | 1 | 45 | 1 | 1 |
| 133 | 33 | 63.00 |  | 1 | 45 | 1 | 1 |
| 134 | 34 | 53.00 |  | 2 | 45 | 1 | 1 |
| 135 | 35 | 65.00 |  | 2 | 45 | 1 | 1 |
| 136 | 36 | 56.00 |  | 2 | 45 | 1 | 1 |
| 137 | 37 | 55.00 |  | 1 | 45 | 1 | 1 |
| 138 | 38 | 32.00 |  | 2 | 45 | 1 | 1 |
| 139 | 39 | 14.00 |  | 1 | 45 | 1 | 1 |
| 140 | 40 | 41.00 |  | 1 | 45 | 1 | 1 |

|  | illness |  | DarkhastM... | elateErja | z  a  k  h  m |  | khoonrizi | tavarom |
| --- | --- | --- | --- | --- | --- | --- | --- | --- |
| 106 |  | 2 | 2 | . |  | 1 | 1 | 2 |
| 107 |  | 2 | 2 | . |  | 2 | 2 | 2 |
| 108 |  | 1 | 2 | . |  | 1 | 2 | 2 |
| 109 |  | 1 | 2 | . |  | 2 | 2 | 2 |
| 110 |  | 1 | 2 | . |  | 2 | 2 | 2 |
| 111 |  | 2 | 2 | . |  | 1 | 2 | 2 |
| 112 |  | 3 | 2 | . |  | 2 | 2 | 2 |
| 113 |  | 1 | 2 | . |  | 1 | 2 | 2 |
| 114 |  | 4 | 2 | . |  | 2 | 2 | 2 |
| 115 |  | 1 | 2 | . |  | 2 | 2 | 2 |
| 116 |  | 3 | 2 | . |  | 2 | 2 | 1 |
| 117 |  | 2 | 2 | . |  | 2 | 2 | 2 |
| 118 |  | 3 | 2 | . |  | 2 | 2 | 2 |
| 119 |  | 1 | 2 | . |  | 2 | 2 | 2 |
| 120 |  | 4 | 2 | . |  | 2 | 2 | 2 |
| 121 |  | 4 | 2 | . |  | 2 | 2 | 2 |
| 122 |  | 2 | 2 | . |  | 2 | 2 | 2 |
| 123 |  | 4 | 2 | . |  | 2 | 2 | 2 |
| 124 |  | 1 | 2 | . |  | 2 | 2 | 2 |
| 125 |  | 2 | 2 | . |  | 2 | 2 | 2 |
| 126 |  | 4 | 2 | . |  | 2 | 2 | 2 |
| 127 |  | 2 | 2 | . |  | 1 | 2 | 2 |
| 128 |  | 2 | 2 | . |  | 2 | 2 | 2 |
| 129 |  | 3 | 2 | . |  | 1 | 2 | 1 |
| 130 |  | 2 | 2 | . |  | 2 | 2 | 2 |
| 131 |  | 2 | 2 | . |  | 2 | 2 | 2 |
| 132 |  | 2 | 2 | . |  | 2 | 2 | 2 |
| 133 |  | 2 | 2 | . |  | 2 | 2 | 2 |
| 134 |  | 3 | 2 | . |  | 2 | 2 | 2 |
| 135 |  | 3 | 2 | . |  | 2 | 2 | 2 |
| 136 |  | 3 | 2 | . |  | 2 | 2 | 2 |
| 137 |  | 3 | 2 | . |  | 2 | 2 | 2 |
| 138 |  | 4 | 2 | . |  | 2 | 2 | 2 |
| 139 |  | 1 | 2 | . |  | 2 | 2 | 2 |
| 140 |  | 5 | 2 | . |  | 2 | 2 | 2 |

|  | dandani | symptom | TazahorSayer | a  n  t  i  m  i  c  r  o  b  i  a  l | zedeEltehab | asareTarkibi |
| --- | --- | --- | --- | --- | --- | --- |
| 106 | 2 | 1 | 2 | 1 | 2 | 1 |
| 107 | 2 | 1 | 2 | 1 | 2 | 2 |
| 108 | 2 | 2 | 2 | 1 | 2 | 2 |
| 109 | 2 | 2 | 2 | 2 | 2 | 2 |
| 110 | 2 | 2 | 2 | 1 | 2 | 1 |
| 111 | 2 | 2 | 2 | 2 | 2 | 2 |
| 112 | 2 | 2 | 2 | 2 | 2 | 2 |
| 113 | 2 | 2 | 2 | 1 | 2 | 1 |
| 114 | 2 | 2 | 2 | 2 | 2 | 2 |
| 115 | 2 | 2 | 2 | 2 | 2 | 2 |
| 116 | 2 | 2 | 2 | 2 | 2 | 2 |
| 117 | 2 | 2 | 1 | 2 | 2 | 2 |
| 118 | 2 | 1 | 2 | 2 | 2 | 2 |
| 119 | 2 | 2 | 2 | 2 | 2 | 2 |
| 120 | 2 | 2 | 2 | 2 | 2 | 2 |
| 121 | 2 | 2 | 2 | 2 | 2 | 2 |
| 122 | 2 | 2 | 2 | 2 | 2 | 2 |
| 123 | 2 | 2 | 2 | 2 | 2 | 2 |
| 124 | 2 | 2 | 2 | 2 | 2 | 2 |
| 125 | 2 | 2 | 2 | 2 | 2 | 2 |
| 126 | 2 | 2 | 2 | 2 | 2 | 2 |
| 127 | 2 | 2 | 2 | 1 | 2 | 2 |
| 128 | 2 | 2 | 2 | 2 | 2 | 2 |
| 129 | 2 | 2 | 2 | 2 | 2 | 2 |
| 130 | 2 | 1 | 2 | 1 | 2 | 1 |
| 131 | 1 | 2 | 2 | 2 | 2 | 1 |
| 132 | 2 | 2 | 2 | 2 | 2 | 2 |
| 133 | 2 | 2 | 1 | 1 | 2 | 1 |
| 134 | 1 | 2 | 2 | 1 | 2 | 2 |
| 135 | 2 | 2 | 2 | 2 | 2 | 2 |
| 136 | 2 | 2 | 2 | 2 | 2 | 2 |
| 137 | 2 | 2 | 2 | 2 | 2 | 2 |
| 138 | 2 | 2 | 2 | 2 | 2 | 2 |
| 139 | 2 | 2 | 2 | 2 | 2 | 2 |
| 140 | 2 | 2 | 2 | 2 | 2 | 2 |

|  | Dastoorola... |
| --- | --- |
| 106 | 2 |
| 107 | 2 |
| 108 | 2 |
| 109 | 2 |
| 110 | 2 |
| 111 | 2 |
| 112 | 2 |
| 113 | 1 |
| 114 | 2 |
| 115 | 2 |
| 116 | 2 |
| 117 | 2 |
| 118 | 2 |
| 119 | 2 |
| 120 | 2 |
| 121 | 2 |
| 122 | 2 |
| 123 | 2 |
| 124 | 2 |
| 125 | 2 |
| 126 | 2 |
| 127 | 2 |
| 128 | 2 |
| 129 | 2 |
| 130 | 2 |
| 131 | 2 |
| 132 | 2 |
| 133 | 2 |
| 134 | 2 |
| 135 | 2 |
| 136 | 2 |
| 137 | 2 |
| 138 | 2 |
| 139 | 2 |
| 140 | 2 |

|  | number | agePatient | gender |  | a  g  e  O  n  c  o  l  o  .  .  . | univOncolo... | bimeh |
| --- | --- | --- | --- | --- | --- | --- | --- |
| 141 | 41 | 16.00 |  | 1 | 45 | 1 | 3 |
| 142 | 42 | 78.00 |  | 2 | 45 | 1 | 3 |
| 143 | 43 | 69.00 |  | 2 | 45 | 1 | 3 |
| 144 | 44 | 54.00 |  | 1 | 45 | 1 | 3 |
| 145 | 45 | 82.00 |  | 1 | 45 | 1 | 3 |
| 146 | 46 | 63.00 |  | 1 | 45 | 1 | 3 |
| 147 | 47 | 51.00 |  | 1 | 45 | 1 | 3 |
| 148 | 48 | 63.00 |  | 1 | 45 | 1 | 2 |
| 149 | 49 | 23.00 |  | 2 | 45 | 1 | 1 |
| 150 | 50 | 60.00 |  | 2 | 45 | 1 | 1 |
| 151 | 51 | 27.00 |  | 1 | 45 | 1 | 1 |
| 152 | 52 | 37.00 |  | 2 | 45 | 1 | 1 |
| 153 | 53 | 37.00 |  | 2 | 45 | 1 | 1 |
| 154 | 54 | 37.00 |  | 2 | 45 | 1 | 1 |
| 155 | 55 | 84.00 |  | 1 | 45 | 1 | 1 |
| 156 | 56 | 37.00 |  | 2 | 45 | 1 | 1 |
| 157 | 57 | 74.00 |  | 1 | 45 | 1 | 1 |
| 158 | 58 | 46.00 |  | 2 | 45 | 1 | 1 |
| 159 | 59 | 25.00 |  | 2 | 45 | 1 | 1 |
| 160 | 60 | 37.00 |  | 2 | 45 | 1 | 1 |
| 161 | ? | ? |  | ? | ? | ? | ? |
| 162 | ? | ? |  | ? | ? | ? | ? |
| 163 | ? | ? |  | ? | ? | ? | ? |
| 164 | ? | ? |  | ? | ? | ? | ? |
| 165 | ? | ? |  | ? | ? | ? | ? |
| 166 | ? | ? |  | ? | ? | ? | ? |
| 167 | ? | ? |  | ? | ? | ? | ? |
| 168 | ? | ? |  | ? | ? | ? | ? |
| 169 | ? | ? |  | ? | ? | ? | ? |
| 170 | ? | ? |  | ? | ? | ? | ? |
| 171 | ? | ? |  | ? | ? | ? | ? |
| 172 | ? | ? |  | ? | ? | ? | ? |
| 173 | ? | ? |  | ? | ? | ? | ? |
| 174 | ? | ? |  | ? | ? | ? | ? |
| 175 | ? | ? |  | ? | ? | ? | ? |

|  | illness |  | DarkhastM... | elateErja | z  a  k  h  m |  | khoonrizi | tavarom |
| --- | --- | --- | --- | --- | --- | --- | --- | --- |
| 141 |  | 1 | 2 | . |  | 2 | 2 | 1 |
| 142 |  | 2 | 2 | . |  | 1 | 1 | 2 |
| 143 |  | 3 | 2 | . |  | 2 | 2 | 1 |
| 144 |  | 3 | 2 | . |  | 2 | 2 | 2 |
| 145 |  | 3 | 2 | . |  | 2 | 2 | 2 |
| 146 |  | 3 | 2 | . |  | 2 | 2 | 2 |
| 147 |  | 3 | 2 | . |  | 1 | 2 | 2 |
| 148 |  | 4 | 2 | . |  | 1 | 2 | 2 |
| 149 |  | 1 | 2 | . |  | 2 | 2 | 2 |
| 150 |  | 1 | 2 | . |  | 2 | 2 | 2 |
| 151 |  | 1 | 2 | . |  | 1 | 2 | 2 |
| 152 |  | 1 | 2 | . |  | 2 | 2 | 2 |
| 153 |  | 1 | 2 | . |  | 2 | 2 | 2 |
| 154 |  | 2 | 2 | . |  | 2 | 2 | 2 |
| 155 |  | 2 | 2 | . |  | 2 | 2 | 2 |
| 156 |  | 2 | 2 | . |  | 2 | 2 | 2 |
| 157 |  | 2 | 2 | . |  | 1 | 2 | 2 |
| 158 |  | 2 | 2 | . |  | 2 | 2 | 2 |
| 159 |  | 2 | 2 | . |  | 2 | 2 | 2 |
| 160 |  | 2 | 2 | . |  | 2 | 2 | 2 |
| 161 |  | ? | ? | ? |  | ? | ? | ? |
| 162 |  | ? | ? | ? |  | ? | ? | ? |
| 163 |  | ? | ? | ? |  | ? | ? | ? |
| 164 |  | ? | ? | ? |  | ? | ? | ? |
| 165 |  | ? | ? | ? |  | ? | ? | ? |
| 166 |  | ? | ? | ? |  | ? | ? | ? |
| 167 |  | ? | ? | ? |  | ? | ? | ? |
| 168 |  | ? | ? | ? |  | ? | ? | ? |
| 169 |  | ? | ? | ? |  | ? | ? | ? |
| 170 |  | ? | ? | ? |  | ? | ? | ? |
| 171 |  | ? | ? | ? |  | ? | ? | ? |
| 172 |  | ? | ? | ? |  | ? | ? | ? |
| 173 |  | ? | ? | ? |  | ? | ? | ? |
| 174 |  | ? | ? | ? |  | ? | ? | ? |
| 175 |  | ? | ? | ? |  | ? | ? | ? |

|  | dandani | symptom | TazahorSayer | a  n  t  i  m  i  c  r  o  b  i  a  l | zedeEltehab | asareTarkibi |
| --- | --- | --- | --- | --- | --- | --- |
| 141 | 2 | 2 | 2 | 2 | 2 | 2 |
| 142 | 2 | 1 | 1 | 2 | 2 | 2 |
| 143 | 2 | 2 | 2 | 2 | 2 | 2 |
| 144 | 2 | 2 | 2 | 2 | 2 | 2 |
| 145 | 2 | 2 | 2 | 2 | 2 | 2 |
| 146 | 2 | 2 | 2 | 2 | 2 | 2 |
| 147 | 2 | 2 | 2 | 2 | 2 | 2 |
| 148 | 2 | 2 | 2 | 1 | 1 | 1 |
| 149 | 2 | 2 | 2 | 2 | 2 | 2 |
| 150 | 2 | 2 | 2 | 2 | 2 | 2 |
| 151 | 2 | 2 | 2 | 2 | 2 | 2 |
| 152 | 2 | 2 | 2 | 2 | 2 | 2 |
| 153 | 2 | 2 | 2 | 2 | 2 | 2 |
| 154 | 2 | 2 | 2 | 2 | 2 | 2 |
| 155 | 2 | 2 | 2 | 1 | 2 | 1 |
| 156 | 2 | 2 | 2 | 1 | 2 | 1 |
| 157 | 2 | 1 | 2 | 2 | 2 | 2 |
| 158 | 2 | 2 | 2 | 2 | 2 | 2 |
| 159 | 2 | 2 | 2 | 2 | 2 | 2 |
| 160 | 2 | 2 | 2 | 2 | 2 | 2 |
| 161 | ? | ? | ? | ? | ? | ? |
| 162 | ? | ? | ? | ? | ? | ? |
| 163 | ? | ? | ? | ? | ? | ? |
| 164 | ? | ? | ? | ? | ? | ? |
| 165 | ? | ? | ? | ? | ? | ? |
| 166 | ? | ? | ? | ? | ? | ? |
| 167 | ? | ? | ? | ? | ? | ? |
| 168 | ? | ? | ? | ? | ? | ? |
| 169 | ? | ? | ? | ? | ? | ? |
| 170 | ? | ? | ? | ? | ? | ? |
| 171 | ? | ? | ? | ? | ? | ? |
| 172 | ? | ? | ? | ? | ? | ? |
| 173 | ? | ? | ? | ? | ? | ? |
| 174 | ? | ? | ? | ? | ? | ? |
| 175 | ? | ? | ? | ? | ? | ? |

|  | Dastoorola... |
| --- | --- |
| 141 | 2 |
| 142 | 2 |
| 143 | 2 |
| 144 | 2 |
| 145 | 2 |
| 146 | 2 |
| 147 | 2 |
| 148 | 2 |
| 149 | 2 |
| 150 | 2 |
| 151 | 2 |
| 152 | 2 |
| 153 | 2 |
| 154 | 2 |
| 155 | 2 |
| 156 | 2 |
| 157 | 2 |
| 158 | 2 |
| 159 | 2 |
| 160 | 2 |
| 161 | ? |
| 162 | ? |
| 163 | ? |
| 164 | ? |
| 165 | ? |
| 166 | ? |
| 167 | ? |
| 168 | ? |
| 169 | ? |
| 170 | ? |
| 171 | ? |
| 172 | ? |
| 173 | ? |
| 174 | ? |
| 175 | ? |

|  | number | agePatient | gender |  | a  g  e  O  n  c  o  l  o  .  .  . | univOncolo... | bimeh |
| --- | --- | --- | --- | --- | --- | --- | --- |
| 176 | ? | ? |  | ? | ? | ? | ? |
| 177 | ? | ? |  | ? | ? | ? | ? |
| 178 | ? | ? |  | ? | ? | ? | ? |
| 179 | ? | ? |  | ? | ? | ? | ? |
| 180 | ? | ? |  | ? | ? | ? | ? |
| 181 | ? | ? |  | ? | ? | ? | ? |
| 182 | ? | ? |  | ? | ? | ? | ? |
| 183 | ? | ? |  | ? | ? | ? | ? |
| 184 | ? | ? |  | ? | ? | ? | ? |
| 185 | ? | ? |  | ? | ? | ? | ? |
| 186 | ? | ? |  | ? | ? | ? | ? |
| 187 | ? | ? |  | ? | ? | ? | ? |
| 188 | ? | ? |  | ? | ? | ? | ? |
| 189 | ? | ? |  | ? | ? | ? | ? |
| 190 | ? | ? |  | ? | ? | ? | ? |
| 191 | ? | ? |  | ? | ? | ? | ? |
| 192 | ? | ? |  | ? | ? | ? | ? |
| 193 | ? | ? |  | ? | ? | ? | ? |
| 194 | ? | ? |  | ? | ? | ? | ? |
| 195 | ? | ? |  | ? | ? | ? | ? |
| 196 | ? | ? |  | ? | ? | ? | ? |
| 197 | ? | ? |  | ? | ? | ? | ? |
| 198 | ? | ? |  | ? | ? | ? | ? |
| 199 | ? | ? |  | ? | ? | ? | ? |
| 200 | ? | ? |  | ? | ? | ? | ? |
| 201 | ? | ? |  | ? | ? | ? | ? |
| 202 | ? | ? |  | ? | ? | ? | ? |
| 203 | ? | ? |  | ? | ? | ? | ? |
| 204 | ? | ? |  | ? | ? | ? | ? |
| 205 | ? | ? |  | ? | ? | ? | ? |
| 206 | ? | ? |  | ? | ? | ? | ? |
| 207 | ? | ? |  | ? | ? | ? | ? |
| 208 | ? | ? |  | ? | ? | ? | ? |
| 209 | ? | ? |  | ? | ? | ? | ? |
| 210 | ? | ? |  | ? | ? | ? | ? |

|  | illness |  | DarkhastM... | elateErja | z  a  k  h  m |  | khoonrizi | tavarom |
| --- | --- | --- | --- | --- | --- | --- | --- | --- |
| 176 |  | 2 | 2 | . |  | 2 | 2 | 2 |
| 177 |  | 2 | 2 | . |  | 2 | 2 | 1 |
| 178 |  | 2 | 2 | . |  | 2 | 2 | 2 |
| 179 |  | 3 | 2 | . |  | 2 | 2 | 2 |
| 180 |  | 3 | 2 | . |  | 2 | 2 | 2 |
| 181 |  | 1 | 2 | . |  | 2 | 2 | 2 |
| 182 |  | 1 | 2 | . |  | 2 | 1 | 2 |
| 183 |  | 1 | 2 | . |  | 2 | 2 | 2 |
| 184 |  | 1 | 2 | . |  | 2 | 2 | 1 |
| 185 |  | 2 | 2 | . |  | 2 | 2 | 2 |
| 186 |  | 4 | 2 | . |  | 2 | 2 | 2 |
| 187 |  | 1 | 2 | . |  | 2 | 1 | 2 |
| 188 |  | 1 | 2 | . |  | 2 | 2 | 1 |
| 189 |  | 2 | 2 | . |  | 2 | 1 | 2 |
| 190 |  | 2 | 2 | . |  | 2 | 2 | 2 |
| 191 |  | 2 | 2 | . |  | 2 | 1 | 2 |
| 192 |  | 3 | 2 | . |  | 2 | 2 | 2 |
| 193 |  | 3 | 2 | . |  | 2 | 1 | 2 |
| 194 |  | 4 | 2 | . |  | 2 | 2 | 2 |
| 195 |  | 2 | 2 | . |  | 2 | 2 | 2 |
| 196 |  | 2 | 2 | . |  | 1 | 2 | 2 |
| 197 |  | 1 | 2 | . |  | 2 | 2 | 1 |
| 198 |  | 2 | 2 | . |  | 2 | 2 | 2 |
| 199 |  | 2 | 2 | . |  | 2 | 1 | 2 |
| 200 |  | 1 | 2 | . |  | 2 | 2 | 2 |
| 201 |  | 2 | 2 | . |  | 2 | 2 | 2 |
| 202 |  | 2 | 2 | . |  | 2 | 2 | 2 |
| 203 |  | 1 | 2 | . |  | 2 | 2 | 2 |
| 204 |  | 1 | 2 | . |  | 2 | 2 | 2 |
| 205 |  | 1 | 2 | . |  | 2 | 2 | 2 |
| 206 |  | 1 | 2 | . |  | 2 | 2 | 2 |
| 207 |  | 1 | 2 | . |  | 2 | 2 | 2 |
| 208 |  | 1 | 2 | . |  | 2 | 2 | 2 |
| 209 |  | 1 | 2 | . |  | 2 | 2 | 2 |
| 210 |  | 1 | 2 | . |  | 2 | 2 | 1 |

|  | dandani | symptom | TazahorSayer | a  n  t  i  m  i  c  r  o  b  i  a  l | zedeEltehab | asareTarkibi |
| --- | --- | --- | --- | --- | --- | --- |
| 176 | 2 | 2 | 2 | 2 | 2 | 2 |
| 177 | 1 | 2 | 2 | 1 | 2 | 1 |
| 178 | 2 | 2 | 2 | 2 | 2 | 2 |
| 179 | 2 | 2 | 2 | 2 | 2 | 2 |
| 180 | 2 | 2 | 2 | 2 | 2 | 2 |
| 181 | 2 | 2 | 2 | 2 | 2 | 2 |
| 182 | 2 | 2 | 2 | 2 | 2 | 1 |
| 183 | 2 | 2 | 2 | 2 | 2 | 2 |
| 184 | 2 | 2 | 2 | 2 | 2 | 2 |
| 185 | 2 | 2 | 2 | 2 | 2 | 2 |
| 186 | 2 | 2 | 2 | 2 | 2 | 2 |
| 187 | 2 | 2 | 2 | 2 | 2 | 2 |
| 188 | 2 | 2 | 2 | 2 | 2 | 2 |
| 189 | 2 | 2 | 2 | 2 | 2 | 2 |
| 190 | 2 | 2 | 2 | 1 | 2 | 2 |
| 191 | 2 | 2 | 2 | 2 | 2 | 2 |
| 192 | 2 | 2 | 2 | 2 | 2 | 2 |
| 193 | 2 | 2 | 2 | 2 | 2 | 2 |
| 194 | 2 | 2 | 2 | 1 | 2 | 2 |
| 195 | 2 | 2 | 2 | 2 | 2 | 2 |
| 196 | 2 | 2 | 2 | 2 | 2 | 2 |
| 197 | 2 | 2 | 2 | 2 | 2 | 2 |
| 198 | 2 | 2 | 2 | 1 | 2 | 2 |
| 199 | 2 | 2 | 2 | 2 | 2 | 2 |
| 200 | 2 | 2 | 2 | 1 | 2 | 2 |
| 201 | 2 | 2 | 2 | 2 | 2 | 2 |
| 202 | 2 | 2 | 2 | 2 | 2 | 2 |
| 203 | 2 | 2 | 2 | 2 | 2 | 2 |
| 204 | 2 | 2 | 2 | 2 | 2 | 2 |
| 205 | 2 | 2 | 2 | 2 | 2 | 2 |
| 206 | 2 | 2 | 2 | 1 | 1 | 1 |
| 19 | 2 | 2 | 2 | 2 | 2 | 2 |
| 208 | 2 | 2 | 2 | 2 | 2 | 1 |
| 209 | 2 | 2 | 2 | 2 | 2 | 2 |
| 210 | 2 | 2 | 2 | 2 | 2 | 2 |

|  | Dastoorola... |
| --- | --- |
| 176 | 2 |
| 177 | 2 |
| 178 | 2 |
| 179 | 2 |
| 180 | 2 |
| 181 | 2 |
| 182 | 2 |
| 183 | 2 |
| 184 | 2 |
| 185 | 2 |
| 186 | 2 |
| 187 | 2 |
| 188 | 2 |
| 189 | 2 |
| 190 | 2 |
| 191 | 2 |
| 192 | 2 |
| 193 | 2 |
| 194 | 2 |
| 195 | 2 |
| 196 | 2 |
| 197 | 2 |
| 198 | 2 |
| 199 | 2 |
| 200 | 2 |
| 201 | 2 |
| 202 | 2 |
| 203 | 2 |
| 204 | 2 |
| 205 | 2 |
| 206 | 1 |
| 207 | 2 |
| 208 | 2 |
| 209 | 2 |
| 210 | 2 |

|  | number | agePatient | gender | a  g  e  O  n  c  o  l  o  .  .  . | univOncolo... | bimeh |
| --- | --- | --- | --- | --- | --- | --- |
| 211 | 15 | 3.00 | 1 | 2 | 1 | 2 |
| 212 | 2 | 3.00 | 1 | 51 | 1 | 2 |
| 213 | 1 | 3.00 | 44 | 24 | 2 | 1 |
| 214 | 14 | 3.00 | 1 | 51 | 3 | 2 |
| 215 | 2 | 6.00 | 1 | 51 | 3 | 2 |
| 216 | 16 | 7.00 | 1 | 51 | 3 | 2 |
| 217 | 17 | 9.00 | 1 | 51 | 3 | 2 |
| 218 | 18 | 3.00 | 1 | 51 | 3 | 2 |
| 219 | 19 | 5.00 | 2 | 51 | 3 | 1 |
| 220 | 20 | 4.00 | 1 | 51 | 3 | 1 |
| 221 | 21 | 5.00 | 2 | 51 | 3 | 1 |
| 222 | 22 | 3.00 | 2 | 51 | 3 | 1 |
| 223 | 23 | 10.00 | 2 | 51 | 3 | 1 |
| 224 | 24 | 8.00 | 1 | 51 | 3 | 1 |
| 225 | 25 | 4.00 | 2 | 51 | 3 | 1 |
| 226 | 26 | 2.00 | 2 | 51 | 3 | 1 |
| 227 | 27 | 7.00 | 1 | 51 | 3 | 1 |
| 228 | 28 | 4.00 | 2 | 51 | 3 | 1 |
| 229 | 29 | 5.00 | 2 | 51 | 3 | 1 |
| 230 | 30 | 8.00 | 1 | 51 | 3 | 1 |
| 231 | 31 | 5.00 | 2 | 51 | 3 | 1 |
| 232 | 32 | 5.00 | 1 | 51 | 3 | 1 |
| 233 | 33 | 9.00 | 2 | 51 | 3 | 1 |
| 234 | 34 | 7.00 | 1 | 51 | 3 | 1 |
| 235 | 35 | 12.00 | 1 | 51 | 3 | 1 |
| 236 | 36 | 6.00 | 2 | 51 | 3 | 1 |
| 237 | 37 | 5.00 | 2 | 51 | 3 | 1 |
| 238 | 38 | 4.00 | 1 | 51 | 3 | 1 |
| 239 | 39 | 3.00 | 2 | 51 | 3 | 1 |
| 240 | 40 | 4.00 | 1 | 51 | 3 | 1 |
| 241 | 41 | 3.00 | 1 | 51 | 3 | 1 |
| 242 | 42 | 8.00 | 1 | 51 | 3 | 1 |
| 243 | 43 | 4.00 | 2 | 51 | 3 | 1 |
| 244 | 44 | 5.00 | 1 | 51 | 3 | 1 |
| 245 | 45 | 4.00 | 1 | 51 | 3 | 1 |

|  | illness |  | DarkhastM... | elateErja | z  a  k  h  m |  | khoonrizi | tavarom |
| --- | --- | --- | --- | --- | --- | --- | --- | --- |
| 211 |  | 1 | 2 | . |  | 1 | 2 | 2 |
| 212 |  | 1 | 2 | . |  | 2 | 2 | 2 |
| 213 |  | 1 | 2 | . |  | 2 | 2 | 2 |
| 214 |  | 1 | 2 | . |  | 2 | 1 | 2 |
| 215 |  | 1 | 2 | . |  | 2 | 2 | 2 |
| 216 |  | 1 | 2 | . |  | 2 | 2 | 2 |
| 217 |  | 1 | 2 | . |  | 2 | 2 | 2 |
| 218 |  | 1 | 2 | . |  | 2 | 2 | 1 |
| 219 |  | 1 | 2 | . |  | 2 | 1 | 2 |
| 220 |  | 1 | 2 | . |  | 1 | 2 | 2 |
| 221 |  | 1 | 2 | . |  | 1 | 2 | 2 |
| 222 |  | 1 | 1 | 1 |  | 1 | 2 | 1 |
| 223 |  | 1 | 2 | . |  | 2 | 2 | 2 |
| 224 |  | 1 | 2 | . |  | 1 | 2 | 2 |
| 225 |  | 1 | 1 | 1 |  | 2 | 2 | 1 |
| 226 |  | 1 | 1 | 1 |  | 2 | 2 | 1 |
| 227 |  | 1 | 2 | . |  | 2 | 2 | 2 |
| 228 |  | 1 | 2 | . |  | 2 | 2 | 2 |
| 229 |  | 1 | 2 | . |  | 2 | 2 | 2 |
| 230 |  | 1 | 2 | . |  | 2 | 2 | 2 |
| 231 |  | 1 | 2 | . |  | 1 | 1 | 2 |
| 232 |  | 1 | 2 | . |  | 2 | 2 | 2 |
| 233 |  | 1 | 2 | . |  | 2 | 2 | 2 |
| 234 |  | 1 | 2 | . |  | 2 | 2 | 2 |
| 235 |  | 1 | 2 | . |  | 2 | 2 | 2 |
| 236 |  | 1 | 2 | . |  | 2 | 2 | 2 |
| 237 |  | 1 | 2 | . |  | 2 | 1 | 2 |
| 238 |  | 1 | 2 | . |  | 2 | 2 | 2 |
| 239 |  | 1 | 2 | . |  | 2 | 2 | 2 |
| 240 |  | 1 | 2 | . |  | 2 | 2 | 1 |
| 241 |  | 1 | 2 | . |  | 2 | 2 | 2 |
| 242 |  | 1 | 2 | . |  | 2 | 2 | 2 |
| 243 |  | 1 | 1 | 1 |  | 2 | 1 | 2 |
| 244 |  | 1 | 2 | . |  | 2 | 2 | 2 |
| 245 |  | 1 | 2 | . |  | 2 | 2 | 2 |

|  | dandani | symptom | TazahorSayer | a  n  t  i  m  i  c  r  o  b  i  a  l | zedeEltehab | asareTarkibi |
| --- | --- | --- | --- | --- | --- | --- |
| 211 | 2 | 2 | 2 | 2 | 2 | 2 |
| 212 | 2 | 2 | 2 | 2 | 2 | 1 |
| 213 | 2 | 2 | 2 | 2 | 2 | 2 |
| 214 | 2 | 2 | 2 | 2 | 2 | 1 |
| 215 | 2 | 2 | 2 | 2 | 1 | 2 |
| 216 | 2 | 2 | 2 | 2 | 1 | 2 |
| 217 | 2 | 2 | 2 | 2 | 2 | 2 |
| 218 | 2 | 2 | 2 | 2 | 1 | 2 |
| 219 | 2 | 2 | 2 | 2 | 2 | 1 |
| 220 | 2 | 2 | 2 | 1 | 1 | 1 |
| 221 | 2 | 1 | 2 | 2 | 1 | 1 |
| 222 | 1 | 2 | 1 | 1 | 1 | 1 |
| 223 | 2 | 2 | 2 | 2 | 2 | 2 |
| 224 | 2 | 2 | 2 | 2 | 2 | 2 |
| 225 | 1 | 2 | 2 | 1 | 1 | 2 |
| 226 | 1 | 2 | 2 | 1 | 2 | 1 |
| 227 | 2 | 2 | 2 | 2 | 2 | 1 |
| 228 | 2 | 2 | 2 | 2 | 2 | 1 |
| 229 | 2 | 2 | 2 | 2 | 2 | 2 |
| 230 | 2 | 2 | 2 | 2 | 2 | 2 |
| 231 | 2 | 1 | 2 | 1 | 1 | 1 |
| 232 | 2 | 2 | 2 | 2 | 2 | 2 |
| 233 | 2 | 2 | 2 | 2 | 2 | 2 |
| 234 | 2 | 2 | 2 | 2 | 2 | 2 |
| 235 | 2 | 2 | 2 | 2 | 2 | 2 |
| 236 | 2 | 2 | 2 | 2 | 2 | 2 |
| 237 | 2 | 2 | 2 | 2 | 2 | 1 |
| 238 | 2 | 2 | 2 | 2 | 2 | 2 |
| 239 | 2 | 2 | 2 | 2 | 2 | 2 |
| 240 | 2 | 2 | 2 | 2 | 2 | 1 |
| 241 | 2 | 2 | 2 | 2 | 2 | 1 |
| 242 | 2 | 2 | 2 | 2 | 2 | 2 |
| 243 | 2 | 2 | 2 | 2 | 1 | 1 |
| 244 | 2 | 2 | 2 | 2 | 2 | 2 |
| 245 | 2 | 2 | 2 | 2 | 2 | 2 |

|  | Dastoorola... |
| --- | --- |
| 211 | 2 |
| 212 | 1 |
| 213 | 2 |
| 214 | 2 |
| 215 | 2 |
| 216 | 2 |
| 217 | 2 |
| 218 | 2 |
| 219 | 1 |
| 220 | 2 |
| 221 | 1 |
| 222 | 1 |
| 223 | 2 |
| 224 | 2 |
| 225 | 2 |
| 226 | 2 |
| 227 | 2 |
| 228 | 2 |
| 229 | 2 |
| 230 | 2 |
| 231 | 1 |
| 232 | 2 |
| 233 | 2 |
| 234 | 2 |
| 235 | 2 |
| 236 | 2 |
| 237 | 1 |
| 238 | 2 |
| 239 | 2 |
| 240 | 1 |
| 241 | 1 |
| 242 | 2 |
| 243 | 2 |
| 244 | 2 |
| 245 | 2 |

|  | number | agePatient | gender |  | a  g  e  O  n  c  o  l  o  .  .  . | univOncolo... | bimeh |
| --- | --- | --- | --- | --- | --- | --- | --- |
| 246 | 46 | 5.00 |  | 2 | 51 | 3 | 1 |
| 247 | 47 | 3.00 |  | 2 | 51 | 3 | 1 |
| 248 | 48 | 8.00 |  | 2 | 51 | 3 | 1 |
| 249 | 49 | 8.00 |  | 2 | 51 | 3 | 1 |
| 250 | 50 | 1.00 |  | 1 | 51 | 3 | 1 |
| 251 | 51 | 9.00 |  | 1 | 51 | 3 | 1 |
| 252 | 52 | 4.00 |  | 2 | 51 | 3 | 1 |
| 253 | 53 | 8.00 |  | 1 | 51 | 3 | 1 |
| 254 | 54 | 3.00 |  | 1 | 51 | 3 | 1 |
| 255 | 55 | 3.00 |  | 2 | 51 | 3 | 1 |
| 256 | 56 | 8.00 |  | 1 | 51 | 3 | 1 |
| 257 | 57 | 13.00 |  | 2 | 51 | 3 | 1 |
| 258 | 58 | 8.00 |  | 1 | 51 | 3 | 1 |
| 259 | 59 | 7.00 |  | 1 | 51 | 3 | 1 |
| 260 | 60 | 5.00 |  | 2 | 51 | 3 | 1 |
| 261 | 61 | 7.00 |  | 1 | 51 | 3 | 1 |
| 262 | 62 | 2.00 |  | 2 | 51 | 3 | 1 |
| 263 | 63 | 6.00 |  | 1 | 51 | 3 | 1 |
| 264 | 64 | 5.00 |  | 1 | 51 | 3 | 1 |
| 265 | 65 | 10.00 |  | 2 | 51 | 3 | 1 |
| 266 | 66 | 9.00 |  | 2 | 51 | 3 | 1 |
| 267 | 67 | 5.00 |  | 2 | 51 | 3 | 1 |
| 268 | 68 | 4.00 |  | 1 | 51 | 3 | 1 |
| 269 | 69 | 2.00 |  | 1 | 51 | 3 | 1 |
| 270 | 70 | 4.00 |  | 2 | 51 | 3 | 1 |
| 271 | 71 | 6.00 |  | 1 | 51 | 3 | 7 |
| 272 | 72 | 4.00 |  | 2 | 51 | 3 | 3 |
| 273 | 73 | 11.00 |  | 2 | 51 | 3 | 3 |
| 274 | 74 | 6.00 |  | 1 | 51 | 3 | 3 |
| 275 | 75 | 6.00 |  | 1 | 51 | 3 | 3 |
| 276 | 76 | 2.00 |  | 1 | 51 | 3 | 3 |
| 277 | 77 | 5.00 |  | 1 | 51 | 3 | 3 |
| 278 | 78 | 8.00 |  | 2 | 51 | 3 | 3 |
| 279 | 79 | 13.00 |  | 1 | 51 | 3 | 3 |
| 280 | 80 | 5.00 |  | 2 | 51 | 3 | 3 |

|  | illness |  | DarkhastM... | elateErja | z  a  k  h  m |  | khoonrizi | tavarom |
| --- | --- | --- | --- | --- | --- | --- | --- | --- |
| 246 |  | 1 | 2 | . |  | 2 | 2 | 2 |
| 247 |  | 1 | 2 | . |  | 2 | 2 | 2 |
| 248 |  | 1 | 2 | . |  | 2 | 2 | 2 |
| 249 |  | 1 | 2 | . |  | 2 | 2 | 2 |
| 250 |  | 1 | 2 | . |  | 2 | 2 | 2 |
| 251 |  | 1 | 2 | . |  | 2 | 2 | 2 |
| 252 |  | 1 | 1 | 1 |  | 2 | 1 | 2 |
| 253 |  | 1 | 2 | . |  | 2 | 2 | 2 |
| 254 |  | 1 | 2 | . |  | 2 | 2 | 2 |
| 255 |  | 1 | 2 | . |  | 2 | 2 | 1 |
| 256 |  | 1 | 2 | . |  | 1 | 2 | 2 |
| 257 |  | 1 | 2 | . |  | 2 | 2 | 2 |
| 258 |  | 1 | 2 | . |  | 2 | 2 | 2 |
| 259 |  | 1 | 2 | . |  | 1 | 2 | 2 |
| 260 |  | 1 | 2 | . |  | 2 | 2 | 2 |
| 261 |  | 1 | 2 | . |  | 2 | 2 | 2 |
| 262 |  | 1 | 2 | . |  | 2 | 1 | 2 |
| 263 |  | 1 | 2 | . |  | 2 | 2 | 2 |
| 264 |  | 1 | 2 | . |  | 2 | 2 | 2 |
| 265 |  | 1 | 2 | . |  | 2 | 2 | 2 |
| 266 |  | 1 | 2 | . |  | 2 | 2 | 2 |
| 267 |  | 1 | 1 | 1 |  | 1 | 2 | 2 |
| 268 |  | 1 | 2 | . |  | 2 | 2 | 2 |
| 269 |  | 2 | 2 | . |  | 2 | 2 | 2 |
| 270 |  | 4 | 2 | . |  | 2 | 2 | 2 |
| 271 |  | 1 | 2 | . |  | 1 | 2 | 1 |
| 272 |  | 1 | 2 | . |  | 2 | 2 | 2 |
| 273 |  | 1 | 2 | . |  | 2 | 2 | 2 |
| 274 |  | 1 | 2 | . |  | 2 | 2 | 2 |
| 275 |  | 1 | 2 | . |  | 2 | 2 | 2 |
| 276 |  | 1 | 2 | . |  | 2 | 2 | 2 |
| 277 |  | 1 | 2 | . |  | 1 | 2 | 2 |
| 278 |  | 1 | 2 | . |  | 2 | 2 | 2 |
| 279 |  | 1 | 2 | . |  | 2 | 2 | 2 |
| 280 |  | 1 | 2 | . |  | 2 | 2 | 2 |

|  | dandani | symptom | TazahorSayer | a  n  t  i  m  i  c  r  o  b  i  a  l | zedeEltehab | asareTarkibi |
| --- | --- | --- | --- | --- | --- | --- |
| 246 | 2 | 2 | 2 | 2 | 1 | 1 |
| 247 | 2 | 2 | 2 | 2 | 2 | 2 |
| 248 | 2 | 2 | 2 | 2 | 2 | 2 |
| 249 | 1 | 2 | 2 | 2 | 2 | 2 |
| 250 | 2 | 2 | 2 | 2 | 2 | 1 |
| 251 | 2 | 2 | 2 | 2 | 2 | 2 |
| 252 | 2 | 2 | 2 | 2 | 2 | 1 |
| 253 | 2 | 2 | 2 | 2 | 2 | 2 |
| 254 | 2 | 2 | 2 | 2 | 2 | 1 |
| 255 | 2 | 2 | 2 | 2 | 1 | 1 |
| 256 | 2 | 2 | 2 | 2 | 2 | 2 |
| 257 | 2 | 2 | 2 | 2 | 2 | 2 |
| 258 | 2 | 2 | 2 | 2 | 2 | 1 |
| 259 | 2 | 2 | 2 | 2 | 2 | 2 |
| 260 | 2 | 2 | 2 | 2 | 2 | 2 |
| 261 | 2 | 2 | 2 | 2 | 2 | 2 |
| 262 | 2 | 2 | 2 | 2 | 2 | 2 |
| 263 | 2 | 2 | 2 | 2 | 2 | 2 |
| 264 | 2 | 2 | 2 | 2 | 1 | 2 |
| 265 | 2 | 2 | 2 | 2 | 2 | 2 |
| 266 | 2 | 2 | 2 | 2 | 2 | 2 |
| 267 | 2 | 2 | 1 | 2 | 2 | 2 |
| 268 | 2 | 2 | 2 | 1 | 1 | 1 |
| 269 | 2 | 2 | 2 | 1 | 1 | 1 |
| 270 | 2 | 2 | 2 | 2 | 2 | 2 |
| 271 | 2 | 2 | 2 | 2 | 2 | 1 |
| 272 | 2 | 2 | 2 | 2 | 2 | 1 |
| 273 | 2 | 2 | 2 | 1 | 2 | 2 |
| 274 | 2 | 2 | 2 | 2 | 2 | 2 |
| 275 | 2 | 2 | 2 | 2 | 1 | 1 |
| 276 | 2 | 2 | 2 | 2 | 2 | 2 |
| 277 | 2 | 2 | 2 | 2 | 2 | 2 |
| 278 | 2 | 2 | 2 | 2 | 2 | 2 |
| 279 | 2 | 2 | 2 | 2 | 1 | 2 |
| 280 | 2 | 2 | 2 | 2 | 2 | 2 |

|  | Dastoorola... |
| --- | --- |
| 246 | 2 |
| 247 | 2 |
| 248 | 2 |
| 249 | 2 |
| 250 | 2 |
| 251 | 2 |
| 252 | 1 |
| 253 | 2 |
| 254 | 1 |
| 255 | 2 |
| 256 | 2 |
| 257 | 2 |
| 258 | 2 |
| 259 | 2 |
| 260 | 2 |
| 261 | 2 |
| 262 | 2 |
| 263 | 2 |
| 264 | 2 |
| 265 | 2 |
| 266 | 2 |
| 267 | 2 |
| 268 | 2 |
| 269 | 2 |
| 270 | 2 |
| 271 | 2 |
| 272 | 2 |
| 273 | 1 |
| 274 | 1 |
| 275 | 2 |
| 276 | 2 |
| 277 | 2 |
| 278 | 2 |
| 279 | 2 |
| 280 | 2 |

|  | number | agePatient | gender |  | a  g  e  O  n  c  o  l  o  .  .  . | univOncolo... | bimeh |
| --- | --- | --- | --- | --- | --- | --- | --- |
| 281 | 81 | 3.00 |  | 1 | 51 | 3 | 3 |
| 282 | 82 | 8.00 |  | 1 | 51 | 3 | 3 |
| 283 | 83 | 10.00 |  | 2 | 51 | 3 | 7 |
| 284 | 84 | 11.00 |  | 1 | 51 | 3 | 7 |
| 285 | 85 | 11.00 |  | 1 | 51 | 3 | 6 |
| 286 | 86 | 3.00 |  | 1 | 51 | 3 | 6 |
| 287 | 87 | 11.00 |  | 1 | 51 | 3 | 6 |
| 288 | 88 | 10.00 |  | 1 | 51 | 3 | 6 |
| 289 | 89 | 5.00 |  | 2 | 51 | 3 | 6 |
| 290 | 90 | 11.00 |  | 2 | 51 | 3 | 7 |
| 291 | 91 | 12.00 |  | 2 | 51 | 3 | 7 |
| 292 | 92 | 7.00 |  | 2 | 51 | 3 | 5 |
| 293 | 93 | 8.00 |  | 1 | 51 | 3 | 5 |
| 294 | 94 | 9.00 |  | 1 | 51 | 3 | 5 |
| 295 | 95 | 11.00 |  | 1 | 51 | 3 | 5 |
| 296 | 96 | 6.00 |  | 1 | 51 | 3 | 5 |
| 297 | 97 | 8.00 |  | 2 | 51 | 3 | 5 |
| 298 | 98 | 7.00 |  | 2 | 51 | 3 | 5 |
| 299 | 99 | 6.00 |  | 1 | 51 | 3 | 7 |
| 300 | 300 | 3.00 |  | 1 | 51 | 3 | 3 |

|  | illness |  | DarkhastM... | elateErja | z  a  k  h  m |  | khoonrizi | tavarom |
| --- | --- | --- | --- | --- | --- | --- | --- | --- |
| 281 |  | 1 | 2 | . |  | 2 | 2 | 2 |
| 282 |  | 1 | 2 | . |  | 2 | 2 | 2 |
| 283 |  | 1 | 2 | . |  | 2 | 2 | 2 |
| 284 |  | 4 | 2 | . |  | 2 | 2 | 2 |
| 285 |  | 1 | 2 | . |  | 2 | 2 | 2 |
| 286 |  | 1 | 2 | . |  | 2 | 2 | 2 |
| 287 |  | 1 | 2 | . |  | 2 | 2 | 2 |
| 288 |  | 1 | 2 | . |  | 2 | 2 | 2 |
| 289 |  | 1 | 2 | . |  | 2 | 2 | 1 |
| 290 |  | 1 | 2 | . |  | 2 | 2 | 2 |
| 291 |  | 1 | 2 | . |  | 1 | 2 | 2 |
| 292 |  | 1 | 2 | . |  | 2 | 2 | 2 |
| 293 |  | 1 | 2 | . |  | 2 | 2 | 2 |
| 294 |  | 1 | 2 | . |  | 2 | 2 | 2 |
| 295 |  | 1 | 2 | . |  | 2 | 2 | 2 |
| 296 |  | 1 | 2 | . |  | 2 | 2 | 2 |
| 297 |  | 1 | 2 | . |  | 2 | 2 | 2 |
| 298 |  | 1 | 2 | . |  | 2 | 2 | 2 |
| 299 |  | 1 | 2 | . |  | 2 | 2 | 1 |
| 300 |  | 1 | 2 | . |  | 2 | 2 | 2 |

|  | dandani | symptom | TazahorSayer | a  n  t  i  m  i  c  r  o  b  i  a  l | zedeEltehab | asareTarkibi |
| --- | --- | --- | --- | --- | --- | --- |
| 281 | 2 | 2 | 2 | 2 | 2 | 1 |
| 282 | 2 | 2 | 2 | 1 | 1 | 1 |
| 283 | 2 | 2 | 2 | 2 | 2 | 2 |
| 284 | 2 | 2 | 2 | 2 | 2 | 2 |
| 285 | 2 | 2 | 2 | 2 | 2 | 1 |
| 286 | 2 | 2 | 2 | 2 | 1 | 2 |
| 287 | 2 | 1 | 2 | 2 | 2 | 1 |
| 288 | 2 | 2 | 2 | 2 | 2 | 2 |
| 289 | 2 | 2 | 2 | 1 | 1 | 2 |
| 290 | 2 | 2 | 2 | 2 | 2 | 2 |
| 291 | 2 | 2 | 2 | 2 | 2 | 2 |
| 292 | 2 | 2 | 2 | 2 | 2 | 2 |
| 293 | 2 | 2 | 2 | 2 | 2 | 2 |
| 294 | 2 | 2 | 2 | 2 | 2 | 2 |
| 295 | 2 | 2 | 2 | 2 | 2 | 2 |
| 296 | 2 | 2 | 2 | 2 | 1 | 2 |
| 297 | 2 | 2 | 2 | 2 | 1 | 2 |
| 298 | 2 | 2 | 2 | 2 | 1 | 2 |
| 299 | 2 | 2 | 2 | 2 | 2 | 1 |
| 300 | 2 | 2 | 2 | 2 | 2 | 1 |

|  | Dastoorola... |
| --- | --- |
| 281 | 1 |
| 282 | 1 |
| 283 | 2 |
| 284 | 2 |
| 285 | 1 |
| 286 | 2 |
| 287 | 1 |
| 288 | 2 |
| 289 | 2 |
| 290 | 2 |
| 291 | 2 |
| 292 | 2 |
| 293 | 2 |
| 294 | 2 |
| 295 | 2 |
| 296 | 2 |
| 297 | 2 |
| 298 | 2 |
| 299 | 1 |
| 300 | 1 |
